# Staged HIV transmission and treatment in a dynamic model with long-term partnerships

**DOI:** 10.1007/s00285-023-01885-w

**Published:** 2023-04-13

**Authors:** Katharine Gurski, Kathleen Hoffman

**Affiliations:** 1 Department of Mathematics, Howard University, Washington, DC 20059 USA; 2 Department of Mathematics and Statistics, University of Maryland Baltimore County, Baltimore, MD 21250 USA

**Keywords:** HIV, HAART treatment, Viral suppression, Acute, Chronic, Long-term partnerships, 92D30

## Abstract

The transmission dynamics of HIV are closely tied to the duration and overlap of sexual partnerships. We develop an autonomous population model that can account for the possibilities of an infection from either a casual sexual partner or a long-term partner who was either infected at the start of the partnership or has been newly infected since the onset of the partnership. The impact of the long-term partnerships on the rate of infection is captured by calculating the expected values of the rate of infection from these extended contacts. The model includes three stages of infectiousness: acute, chronic, and virally suppressed. We calculate HIV incidence and the fraction of new infections attributed to casual contacts and long-term partnerships allowing for variability in condom usage, the effect of achieving and maintaining viral suppression, and early intervention by beginning HAART during the acute phase of infection. We present our results using data on MSM HIV transmission from the CDC in the U.S. While the acute stage is the most infectious, the majority of the new infections will be transmitted by long-term partners in the chronic stage when condom use is infrequent as is common in long-term relationships. Time series analysis of the solution, as well as parameter sensitivity analysis, are used to determine effective intervention strategies.

## Introduction

In 2019, according to the Center for Disease Control (CDC), 36,801 people received an HIV diagnosis in the United States (US) and dependent areas (Centers for Disease Control and Prevention 2021) and in 2020 over 37 million people globally were living with HIV (UNAIDS 2021). Much scientific effort has been devoted to studying all aspects of HIV infection, from the dynamics of the disease progression in the body to the epidemiology of the spread of the virus. More recent advances in disease progression have asserted three stages: the acute stage (sometimes subdivided into acute and early infectious periods, but in this study these substages are combined), the chronic stage, and finally the AIDS stage (Hernandez-Vargas and Middleton 2013). The acute stage of HIV begins with infection and continues for approximately 8–12 weeks (Fiebig et al. 2003; Robb and Ananworanich 2016). During the acute stage, those infected experience a high viral load and are very contagious. After the acute stage, those infected move into a chronic stage in which their viral load goes down and many individuals are asymptomatic. Although the viral load decreases, the disease can still be transmitted during this stage of the infection. Those who have started highly active antiretroviral therapy (HAART) treatment may be *virally suppressed*, which also decreases the chance of HIV transmission (Cohen et al. 2011a; Granich et al. 2009). The chronic stage of infection may last decades, particularly for those in HAART treatment. At the end of the chronic stage, the viral load begins to rise, and the CD4 cell count begins to drop. When the CD4 cell count drops to below $200 \, \text {cells}/\text {mm}^3$, a person is diagnosed with AIDS, the final stage of the disease. At this stage of the disease, the immune system is severely damaged, which leads to opportunistic infections with increasing frequency. In this model, we are not using AIDS as the new CDC category as first onset of AIDS (Poorolajal et al. 2016), from which individuals may recover using HAART treatment. Instead, we are using the AIDS category for those individuals whose health has declined significantly and are likely to die within 2 years from opportunistic infections.

Unlike the flu, chickenpox, measles and SARS-Cov2 that are transmitted through tangential casual contact (Centers for Disease Control and Prevention 2022b, c, d, e), sexually transmitted diseases require pair formation for transmission. The length of the pair formation partnership may be short, such as a casual partnership, or long, as with long-term partnerships that last weeks, months or even decades. Much literature has been devoted to partnership formation models for sexually transmitted diseases in which the dynamics of pair formation and dissolution are explicitly accounted for in the model (for example, Kretzschmar and Heijne (2017)). These model require moment closure methods to incorporate overlapping partnerships. Recently, Gurski (2019) developed a population model that accounts for overlapping casual and long-term partnerships using a linearization of the expected value as the rate of infection without moment closure methods. In Gurski et al. (2023), pair formation models of Kretzschmar and Heijne (2017) and Leng and Keeling (2018) were compared to a long-term *SI* population of model of HIV and HSV-2. For each model and each disease, the reproduction number was analytically determined and numerically computed over a range of parameter values. Results demonstrated that reproduction numbers and time series simulations of disease dynamics are almost identical in the case without concurrent partnerships. (We made no effort to simulate pair formation models with moment closure methods, so no comparison was available.) Thus, we focus on the autonomous population model of Gurski (2019).

We model three different stages of infections: acute, chronic, and virally suppressed. Each stage of infection has a different level of infectivity with the acute stage being the highest level of infectivity, the chronic stage being less transmissible than the acute stage but more transmissible than the virally suppressed stage. Although the acute stage of infection is the most transmissible, infected individuals only remain in the acute stage for about three months before moving onto the chronic stage or the virally suppressed stage of infection. Since there is no cure yet for HIV, infected individuals remain in either chronic or virally suppressed stage for the rest of their lives and never return to the acute stage. Given the increased transmissibility in the acute stage, there has been much speculation regarding the role of acutely infected individuals in the spread of HIV given the small time span individuals remain in the acute stage. Percentages of new infections caused by transmission from individuals in the acute stage of infection vary from 0% to 93% (Miller et al. 2010) depending on the type of model (casual, long-term partnerships, and concurrent partnerships), the population being modeled (MSM or heterosexual), delineations of subpopulations by culture, type of sexual behavior, and degree of infectiousness (see for example, Miller et al. 2010; Bellan et al. 2015; Omondi et al. 2018; Jacquez et al. 1994; Koopman et al. 1997; Pinkerton and Abramson 1996; Kretzschmar and Dietz 1998; Xiridou et al. 2003, 2004; Hollingsworth et al. 2008; Coutinho et al. 2001; Pinkerton 2007; Hayes and White 2006). We focus on the MSM population using data from the CDC in the USA with both casual and long-term partnerships. While others have focused on explicit partnership models, we instead use a long-term partnership model that incorporates the memory of the partnership into the force of infection term using an expected value. Results from our model show that chronically infected individuals in long-term partnerships contribute to the spread of HIV significantly more than any other population. We find acutely infected individuals in long-term partnerships contribute on average 20% of the new infections, which is consistent with the lower end of Jacquez et al. (1994); Koopman et al. (1997); Pinkerton and Abramson (1996) and the middle of the range suggested by Xiridou et al. (2003, 2004).

Concurrent partnerships account for the possibilities of overlapping partnerships and infection from either a new sexual partner or a non-monogamous longtime partner who was uninfected at the start of the partnership. Concurrency has also been shown to be important in modeling the disproportionate growth of HIV infections in subpopulations (Adimora et al. 2002). The impact of long-term partnerships and concurrent partnerships has been the focus of many data-driven models ranging from Monte Carlo simulations (Kretzschmar and Morris 1996), stochastic simulations (Morris and Kretzschmar 1997; Doherty et al. 2006; Aralis et al. 2016), agent based models (Gopalappa et al. 2017), stochastic and discrete simulations (Chick et al. 2000), network simulations (Admiraal and Handcock 2016; Eames and Keeling 2004; Keeling and Eames 2005; Lashari and Trapman 2018; Morris et al. 2009, 2010; Onaga et al. 2017; Volz and Meyers 2007; Vajdi et al. 2020) and analytic network models (Miller and Slim 2017). Other combinations of statistical and population models have been developed to capture concurrency effects using a partnership-based concurrency index (Leung et al. 2012, 2017), pair-formation models with stochastic pair approximation techniques (Hansson et al. 2019; Kim 2015) and nested pair formation models (Kretzschmar and Heijne 2017; Leng and Keeling 2018). These models, which can include long-term partnerships, have difficulty representing infection from overlapping partnerships, and for each population class, the model must contain sub-populations of each single or pair combination. As a result, the computational and analytical complexity of the model quickly increases, and hence decreases the length of time a longitudinal simulation can be run. In contrast, non-pair population based models do not require the same computational resources and allow for analytic studies.

A deterministic model of concurrency was derived previously in Gurski and Hoffman (2016), following the work of Watts and May (1992) and more recently, Gurski (2019) developed a population model that accounts for the possibilities of an infection from either a casual sexual partner or a long-term partner who was either infected at the start of the partnership or newly infected. The model allows for multiple long-term partnerships, which include serially monogamous and concurrent partnerships. The impact of the long-term partnerships on the rate of infection is captured by calculating the expected values of these extended contacts. The model benefits from the traditional strengths of computational speed and an analytic reproduction number, which in turn allows for understanding of how each parameter affects the disease spread. In addition, both long-term and casual partnerships can be included in the model without moment closure methods, which have been used as a technique for including concurrency. This model was compared to pair formation models in Gurski et al. (2023) demonstrating that, in the absence of concurrency, the long-term partnership model mimics the pair formation model with evaluations of the reproduction number and numerical simulations.

We incorporate the new long-term and casual partnership model (Gurski 2019) into a previous population model described in Gurski and Hoffman (2016) to include both long-term partnerships and three classes of infectiousness: the acute class, $I_a$, chronic class, $I_c$, and virally suppressed class, $I_v$. The populations $I_a$, $I_c$, and $I_v$ can be seen as a three-class infectivity model, a simplified $n-$class infectiousness model (Hyman et al. 1999). In Gurski and Hoffman (2016), the total population was assumed to be constant, which is a reasonable assumption for short timescales. Here, we derive a new deterministic model with concurrency that does not assume a constant population, but alternatively assumes that the U.S. population grows proportional to the population size, at a nearly constant rate (Mackun and Wilson 2011). We also include behavioral disinhibition in the model by reducing the likelihood of condom use by MSM individuals in a long-term partnership.

Incorporating long-term and casual partnerships in a model with differential infectivity allows a novel approach to evaluate intervention strategies that reduce the incidence and prevalence of HIV. The model formulation also allows us to determine which population is the largest contributor to the spread of HIV. In particular, we are interested in determining for short term dynamics, on the order of 20 years, which intervention strategies will have the most impact. We consider three types of intervention strategies.The first intervention is to target primary infection, or acute class infections, for early treatment. While this is not a current emphasis of HIV intervention strategies, partly due to the lack of an accurate easy-to-use test for the acute stage infection, we will show that this intervention might significantly impact the number of new infections. The second intervention strategy is to encourage the chronically infected to achieve and remain in suppression. The impact of this intervention is supported by analysis of the effective reproduction number, time series data, and parameter sensitivity. The third strategy is to target condom effectiveness in long-term relationships. We will demonstrate that the largest percentage of new infections come from partnerships with a long-term chronically infected partner and that increased condom use with long-term partners can impact the incidence of new infections.

The manuscript is organized as follows. Section 2 contains a description of the model with three stages of infection. The derivation of the rate of infection for the model with long-term partnerships and concurrency can be found in Sect. 3. Section 4 contains results including the effective reproduction number and a study of incidence and prevalence where model predictions from 2005–2020 are compared to CDC data. This section also includes numerical results that show which populations are the source of the infection as well as parameter sensitivity studies and the impact of early testing and treatment. Finally, we interpret these results in Sect. 5 with our overall conclusions.

## Model with differential infectiousness

In 1992, an SEIR model for HIV was proposed (Watts and May 1992), where the population was divided into *S*, susceptible; *E*, exposed but not yet infectious; *I*, infected with HIV; and *R*, indicating that the disease has progressed to AIDS and represents removal from the system. Previously, in Gurski and Hoffman (2016) a susceptible-two stage infection model was developed, based on the model of Watts and May (1992), but without the time lag from exposed to infectious, thus removing the exposed class. They included a virally suppressed class and allowed for concurrent partnerships in heterosexual partnerships. One limitation of the model was the lack of an acute class and the absence of serodiscordant long-term partnerships. In Gurski (2019) an autonomous constant sized population model was developed that includes both casual and long-term partnerships, where the impact of the long-term partnerships is included in the rate of infection, which is computed using a linearized expected value. This long-term partnership model includes concurrency without using moment closure methods, to introduce a third party. Serodiscordant long-term partnerships were considered in this case, but included a constant population assumption. This long-term partnership model with concurrency to a two-staged infection SI model for HIV and HSV-2 was expanded in Gurski et al. (2023) with a comparison of their results to a model with two pair formation models. The reproduction number for the long-term partnership model without concurrency is comparable to the pair formation model, demonstrating that, in the absence of concurrency, the long-term partnership model mimics the pair formation model.

**Fig. 1:**
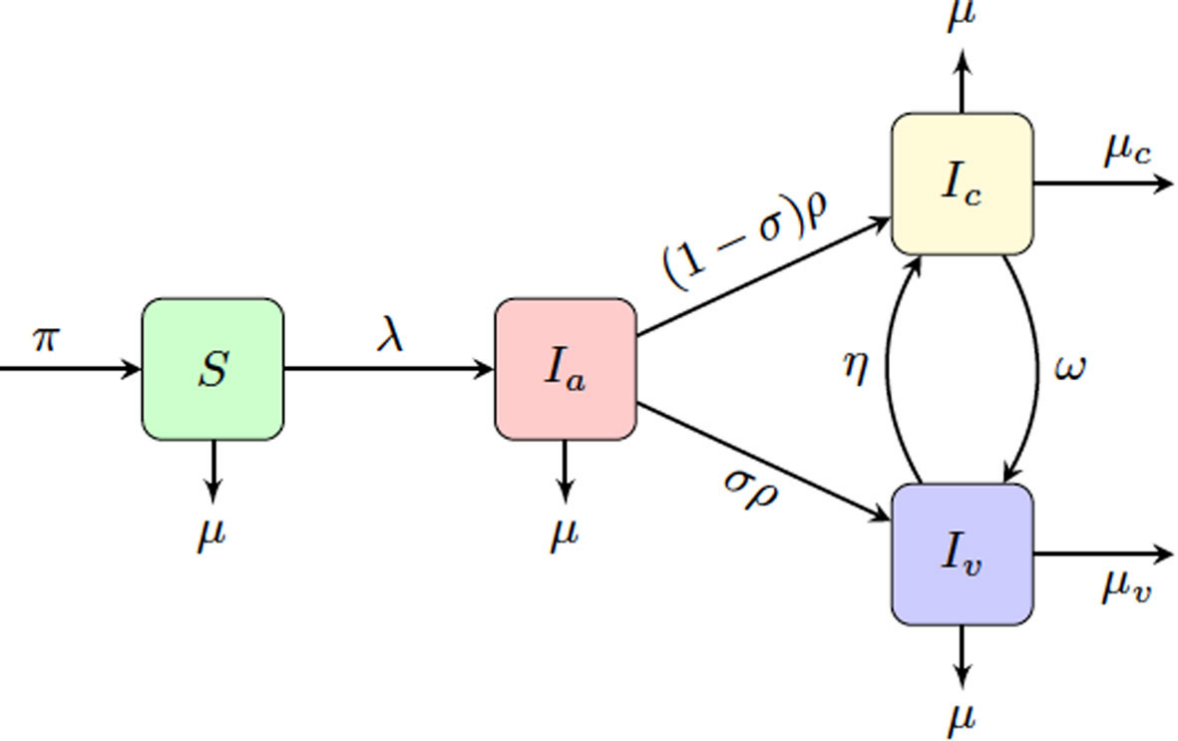
$$S I_a I_c I_v $$ model where *S* is the susceptible population, $$I_a$$ is the acutely infected population, $$I_c$$ is the chronically infected and not virally suppressed population, $$I_v$$ is the infected and virally suppressed population

Here, we modify the previous population model described in Gurski and Hoffman (2016) to include both long-term partnerships and three classes of infectiousness: the acute class, $$I_a$$, chronic class, $$I_c$$, and virally suppressed class, $$I_v$$. This is an $$S I_a I_c I_v $$ model, as illustrated in Fig. 1. The time from onset of infection to the transition to the chronic stage of infection has a length measured to be approximately 10 weeks (Pilcher et al. 2007) to 3 months (Cohen et al. 2011b; Hollingsworth et al. 2008). We refer to this primary infection as the acute class and use a mean infectivity level adjusted to this entire period of increased infectiousness. The primary HIV infection peaks within a month after infection and then slowly declines to a lower viral load associated with a chronic infection.

In this system, *S* represents the susceptible population, $I_a$ represents the population with an acute infection and high infectivity that results at the onset of infection, $I_c$ represents the population with chronic infection that is not virally suppressed, and $I_v$ represents the infected and virally suppressed individuals that are in treatment. We make a distinction between chronically infected and virally suppressed classes because virally suppressed individuals are significantly less likely to transmit HIV to an uninfected partner (Cohen et al. 2011a; Cambiano et al. 2013; Castilla et al. 2005; Porco et al. 2004).

We assume that people become susceptible at rate $\pi$ and leave the susceptible population due to natural death at rate $\mu$. Note that in this model we consider a non-constant population. The susceptible population goes to the acutely infected population at rate $\lambda$. The acutely infected population $I_a$ transition to either the chronically infected population $I_c$ or the virally suppressed population $I_v$ at rate $\rho$. We assume that $\sigma$ is the fraction of the population that goes to virally suppressed, therefore $(1-\sigma )$ is the fraction of the population that will go to the chronically infected population. We note that HIV detection in the acute stage of infection is difficult, hence many undiagnosed acute infections remain untreated. Early treatment has been suggested as an intervention (Granich et al. 2009; Xiridou et al. 2003; Lindback et al. 2000; Ananworanich et al. 2015; Ford et al. 2018), hence we include the parameter in our model.

The chronically infected can move to the virally suppressed population with transition rate $\omega$ and the virally suppressed population can move to the chronically infected population at rate $\eta$. The transition rate to AIDS (AIDS without effective HAART treatment (Poorolajal et al. 2016)) is included as removal from both the chronically infected population $I_c$ and virally suppressed population $I_v$, with rates $\mu _c$ and $\mu _v$, respectively. The total population is $N = S + I_a + I_c + I_v$. The parameter descriptions and range of values are given in Table 1. The set of differential equations that describe the dynamics of these populations are:

1 $$\begin{aligned} \begin{aligned} \frac{dS}{dt}&= \pi - \lambda S - \mu S, \\ \frac{dI_a}{dt}&= \lambda S - \rho I_a - \mu I_a, \\ \frac{d I_c}{dt}&= (1-\sigma ) \rho I_a +\eta I_v -\omega I_c - (\mu +\mu _c) I_c, \\ \frac{d I_v}{dt}&= \sigma \rho I_a -\eta I_v +\omega I_c - (\mu +\mu _v) I_v,\\ \frac{d N}{dt}&= \pi -\mu _c I_c - \mu _v I_v -\mu N. \end{aligned} \end{aligned}$$

## Rates of infection

The rates of infection $\lambda$ depends on the type of partnership. Previous literature focused on explicitly modeling the formation of each pair. Pair formation models explicitly account for the formation and dissolution of pairs and the disease-status of each individual within the pair, however, this detailed modeling requires a large number of differential equations. Our model, on the other hand, includes long-term partnerships using a linearization of the expected value to compute infection rates. The susceptible population can be infected either from a casual partner or a long-term partner. We assume that the transmission rate from an infected individual in population $I_j$ is $z \beta _j$ with $j=a,c,v$ where $\beta _j$ is the transmission probability per sexual encounter with an infected individual in $I_j$, and *z* is the rate of casual sexual encounters. We denote the rate of infection from casual partners $\lambda ^z$ with the standard form:

2 $$\begin{aligned} \lambda ^z = \frac{z \left( \beta _a I_a+ \beta _c I_c +\beta _v I_v\right) }{N}. \end{aligned}$$

The rate of infection from long-term partners is denoted $\lambda ^p$. Infection from long-term partners can come from two sources: a long-term partner who was chosen while infectious, $\lambda ^{pI}$, and a long-term partner who was chosen while susceptible, $\lambda ^{pS}$, but has been newly infected outside the long-term partnership. Thus, the rate of infection $\lambda$ is combination of these rates

$$\begin{aligned} \lambda = \lambda ^z + \lambda ^p, \end{aligned}$$

where

$$\begin{aligned} \lambda ^p = \lambda ^{pI} + \lambda ^{pS}. \end{aligned}$$

We next address the analytical forms of $\lambda ^{pI}$ and $\lambda ^{pS}$.

### Rate of infection due to long-term partner chosen while infectious

Consider the case where a susceptible individual is infected by an infectious long-term partner. We assume that the partnership began at time $\kappa$ and the long-term partner was infected at time *t*. Thus, the rate of transmission is defined to be

3 $$\begin{aligned} \lambda _p^I = E\left[ \frac{\chi _a I_a+ \chi _c I_c + \chi _v I_v}{N}\right] , \end{aligned}$$

where $E\left[ (\chi _a I_a+ \chi _c I_c + \chi _v I_v)/N\right]$ represents the expected value of the rate of infection due to partners initially chosen while infectious, and $\chi _a, \chi _c, \chi _v$ denote the rates of transmission from the three classes of infected individuals $I_a, I_c, I_v$, respectively. We begin the first stage of calculating this expected value by deriving the probability that an infected partner who is acquired at time $\kappa$ transmits the infection at a later time *t* is given by the product of the following probabilities:

A: The probability that a partner acquired at time $\kappa$ was infected at time $\kappa$: $P(X(\kappa ) \in I_a \cup I_c \cup I_v) = \left( I_a(\kappa ) + I_c(\kappa ) + I_v(\kappa )\right) /N(\kappa )$.
B: The probability that a partnership survives until time *t* given that the partner was partner acquired at time $\kappa$ is $e^{ -(t-\kappa )/\tau }$.

Then the expected value becomes

4 $$\begin{aligned} \lambda ^{p I}= & {} E\left[ \frac{\chi _a I_a + \chi _c I_c+ \chi _v I_v}{N}\right] , \nonumber \\= & {} \int _{-\infty }^t \left( \frac{\chi _a I_a (\kappa ) + \chi _c I_c (\kappa ) + \chi _v I_v (\kappa ) }{\tau \, N (\kappa ) }\right) e^{ ( -(t-\kappa )/\tau )} \, d \kappa , \end{aligned}$$

where the parameter $\tau$ represents the mean duration of a partnership. This integral requires us to keep track of the number of infected individuals and total population for all time prior to the instant *t*. To make the calculation algebraically tractable, we will use a linear approximation of $N(\kappa )$. Consider the approximation of the derivative

$$\begin{aligned} \frac{N(t) - N(\kappa )}{t-\kappa } \approx \frac{dN(t)}{dt}, \end{aligned}$$

and substitute $\frac{dN(t)}{dt}=\pi (t) -\mu _c I_c(t) - \mu _v I_v(t) -\mu N(t)$ from (1). We define $\pi (t) \equiv \Lambda N(t)$. Solving for $N(\kappa )$, we get

$$\begin{aligned} N(\kappa )\approx & {} N(t)\left[ 1 + (t-\kappa ) \mu \right] + \left[ \mu _c I_c(t) + \mu _v I_v (t) -\Lambda N(t) \right] (t-\kappa ). \end{aligned}$$

Rearranging this equation to solve for $\frac{1}{N(\kappa )}$, we get

5 $$\begin{aligned} \frac{1}{N(\kappa )}= & {} \frac{1}{[1+(\mu - \Lambda )(t-\kappa )]N(t) + (t-\kappa )[\mu _c I_c + \mu _\nu I_v ]},\nonumber \\= & {} \frac{1}{N(t)} \left[ \frac{1}{1+ (t-\kappa )K_N(t)}\right] , \end{aligned}$$

where

6 $$\begin{aligned} K_N(t) =(\mu -\Lambda ) +\frac{\mu _c I_c(t) + \mu _v I_v(t)}{N(t)} = -\frac{1}{N(t) } \frac{d N(t)}{dt}. \end{aligned}$$

Since $K_N(t) (t-\kappa ) \ll 1$, we approximate $\frac{1}{1+ (t-\kappa )K_N(t) }\approx 1 - (t-\kappa )K_N(t))$, so the expected value (4) becomes

7 $$\begin{aligned} E\left[ \frac{\chi _a I_a + \chi _c I_c + \chi _v I_v}{N}\right]&\approx \frac{1}{\tau N(t)} \int _{-\infty }^t \left( \chi _a I_a (\kappa ) + \chi _c I_c (\kappa ) + \chi _v I_v (\kappa ) \right) \cdot \nonumber \\&\qquad \left( 1 - (t-\kappa )K_N(t) \right) e^{ ( -(t-\kappa )/\tau )} \, d \kappa . \end{aligned}$$

Next we approximate $\chi _a I_a(\kappa ) + \chi _c I_c(\kappa ) + \chi _v I_v(\kappa )$ using the differential equations in (1). Using the same techniques as described above for $N(t)$, we approximate each derivative $\frac{d I_a(t)}{dt}, \frac{d I_c(t)}{dt}, \frac{d I_v(t)}{dt}$. As an example, consider $\frac{d I_a(t)}{dt}$:

8 $$\begin{aligned} \frac{d I_a(t)}{dt} \approx \frac{(I_a(t) - I_a(\kappa )) }{t-\kappa }. \end{aligned}$$

Substituting from (1) for the derivative of $I_a(t)$ and solving for $I_a(\kappa )$, we get

9 $$\begin{aligned} I_a(\kappa ) \approx I_a(t) \left( 1 + (\rho + \mu )\left[ t-\kappa \right] \right) -\lambda S (t) \left[ t-\kappa \right] . \end{aligned}$$

Using a similar technique for $I_c$ and $I_v$ yields the sum

10 $$\begin{aligned}&\chi _a I_a(\kappa ) + \chi _c I_c(\kappa )+ \chi _v I_v(\kappa ) \approx I_a(t) \left( \chi _a + \left\{ \chi _a(\rho + \mu ) -\chi _c (1- \sigma )\rho \right. \right. \nonumber \\&\qquad \left. \left. - \chi _v \sigma \rho \right\} \left[ t-\kappa \right] \right) + I_c(t) \left( \chi _c + \left\{ \chi _c(\mu +\mu _c + \omega ) -\chi _v \omega \right\} \left[ t-\kappa \right] \right) \nonumber \\&\qquad + I_v(t) \left[ \chi _v + \left\{ \chi _v(\mu +\mu _v + \eta ) - \chi _c \eta \right\} (t-\kappa )\right] - \chi _a \lambda S (t)\left[ t-\kappa \right] . \end{aligned}$$

So defining

11 $$\begin{aligned} K_A&\equiv \chi _a (\rho + \mu ) -\chi _c (1- \sigma )\rho - \chi _v \sigma \rho , \nonumber \\ K_C&\equiv \chi _c (\mu +\mu _c + \omega ) -\chi _v \omega ,\nonumber \\ K_V&\equiv \chi _v (\mu +\mu _v + \eta ) - \chi _c \eta , \end{aligned}$$

we have

12 $$\begin{aligned} \chi _a I_a(\kappa ) + \chi _c I_c(\kappa )+ \chi _v I_v(\kappa )&\approx \left( \chi _a+ K_A \left[ t-\kappa \right] \right) I_a (t)+ \nonumber \\&\qquad \left( \chi _c + K_C \left[ t-\kappa \right] \right) I_c(t)+ \left( \chi _v + K_V\left[ t-\kappa \right] ) \right) I_v(t) \nonumber \\&\quad - \chi _a \lambda S(t) \left[ t-\kappa \right] . \end{aligned}$$

Plugging this into the expected value equation (4),

13 $$\begin{aligned} E \left[ \frac{\chi _a I_a + \chi _c I_c + \chi _v I_v}{N} \right]&\approx \frac{1}{\tau N(t)} \int _{-\infty }^t \left[ \left( \chi _a+ K_A \left[ t-\kappa \right] \right) I_a (t)\right. \nonumber \\&\quad \left. + \left( \chi _c + K_C \left[ t-\kappa \right] \right) I_c (t)+ \left( \chi _v + K_V \left[ t-\kappa \right] \right) I_v (t) \right. \nonumber \\&\quad \left. - \chi _a \lambda S(t) \left[ t-\kappa \right] \right] \cdot \left[ 1 - K_N(t) \left[ t-\kappa \right] \right] e^{ ( -(t-\kappa )/\tau )} \, d \kappa , \nonumber \\&\equiv U^I - \lambda \chi _a U^S \equiv \lambda ^{pI} \end{aligned}$$

where

14 $$\begin{aligned} U^I&\equiv \frac{\left( \chi _a I_a(t) + \chi _c I_c(t)+ \chi _v I_v(t) \right) (1 - K_N(t) \tau ) }{N(t)} + \nonumber \\&\qquad \frac{ \left( K_A I_a(t)+ K_C I_c(t)+ K_V I_v(t)\right) \tau (1 - 2 K_N(t) \tau )}{N(t)}, \end{aligned}$$

15 $$\begin{aligned} U^S&\equiv \frac{\tau S (1- 2 K_N(t) \tau )}{N(t)}. \end{aligned}$$

Intuitively, we are approximating the fraction of infectious individuals at time $\kappa$ as the fraction of infected at time *t*, denoted by $U^I$, adding the fraction of infected who died ($\frac{\tau \chi _a S ( 2 K_N(t) \tau )}{N(t)}$), and subtracting the fraction of individuals who became infected between times $\kappa$ and *t*, namely $\frac{\tau \chi _a S}{N(t)}$.

### Derivation of expected value of rate of infection due to long-term partner chosen while susceptible

Next we derive the rate of infection from long-term partners who were susceptible at the start of the partnership, $\lambda ^{pS}$. We assume the infection will be transmitted while the infected partner is in infection class $I_a$ since the new infection is most likely unknown to both partners. In addition, we do not assume that the previously susceptible-susceptible long-term pair use condoms to prevent infection. The rate of infection is

16 $$\begin{aligned} \lambda ^{pS}= E\left[ \frac{\psi I^{new}}{N}\right] , \end{aligned}$$

where $E\left[ \psi I^{new}/N \right]$ represents the expected value of the fraction of newly infected (previously susceptible) partners per total population still in a partnership at time *t*. Again, to keep the model memory free and algebraically tractable we will define $\lambda ^{pS}$ to be a linear approximation to the expected value.

To calculate $I^{new}$, we consider four probabilities:

C: The probability that a partner acquired at time $\kappa$ was susceptible at time $\kappa$: $P(X(\kappa ) \in S) = S(\kappa ) / N(\kappa )$.
D: The probability that a partnership survives until time *t* given that the partner was acquired at time $\kappa$ is $e^{ -(t-\kappa )/\tau }$.
E: The probability that a partner is still susceptible at time *t* given they were susceptible at time $\kappa$: $P((X(t) \in S) \vert (X(\kappa ) \in S)) = e^{-(t-\kappa )\xi \lambda }$, where $\xi$ is the probability that the partner is in engaged in an external (i.e. outside this long-term partnership) sexual partnership.
F: The probability that the partner becomes infected at time *t*, $\lambda (t-\kappa ) \xi$ where $\lambda$ is the rate of infection and $(t-\kappa )$ is the partnership length.

Thus, the rate of transmission is

17 $$\begin{aligned} \lambda ^{pS}= & {} E\left[ \frac{\psi I^{new}_A}{N} \right] , \nonumber \\= & {} \int _{-\infty }^t \left( \frac{\psi \lambda (t-\kappa ) \xi S (\kappa ) }{N (\kappa ) }\right) \left( \frac{1+ \xi \lambda \tau }{\tau }\right) e^{-\frac{(1+ \xi \lambda \tau )(t-\kappa )}{\tau }} d \kappa , \end{aligned}$$

where $\left( \frac{1+ \xi \lambda \tau }{\tau }\right) e^{-\frac{(1+ \xi \lambda _\tau )(t-\kappa )}{\tau }}$ is the probability distribution corresponding to the survival functions that the partnership survives and the partner is still susceptible at time *t*, given they were susceptible at time $\kappa$ (probabilities D and E above). We approximate $S(\kappa )$ using (1),

18 $$\begin{aligned} \frac{d S}{dt}\approx & {} \frac{S(t) - S(\kappa )}{t-\kappa }, \end{aligned}$$

19 $$\begin{aligned} S(\kappa )\approx & {} -\Lambda N(t) \left[ t-\kappa \right] + S(t) \left( 1 + (\lambda + \mu ) \left[ t - \kappa \right] \right) . \end{aligned}$$

Then the expected value (17) becomes

20 $$\begin{aligned} \lambda ^{pS}= & {} \frac{\psi \lambda \xi }{N(t)} \int _{-\infty }^t \left[ t-\kappa \right] \left\{ -\Lambda N(t) \left[ t-\kappa \right] + S(t) \left( 1 + (\lambda + \mu ) \left[ t-\kappa \right] \right) \cdot \right. \nonumber \\{} & {} \left. \left( 1- K_N(t) \left[ t-\kappa \right] \right) \right\} \cdot \left( \frac{1+ \xi \lambda \tau }{\tau }\right) e^{-\frac{(1+ \xi \lambda \tau )(t-\kappa )}{\tau }} d \kappa , \nonumber \\= & {} \frac{ \psi }{N(t)} \left[ F(\xi \lambda \tau ,t)\right] . \end{aligned}$$

Linearizing $F(\xi \lambda \tau ,t)$ about $\xi \lambda \tau$, we have

21 $$\begin{aligned} F(\xi \lambda \tau ,t)&\approx \left[ -2 \Lambda N(t) \tau \left( 1-3K_N(t) \tau \right) + S(t) \left\{ \left( 1 - 2 K_N(t) \tau \right) + \right. \right. \nonumber \\&\quad \left. \left. 2 \mu \tau \left( 1-3K_N(t) \tau \right) \right\} \right] \xi \lambda \tau . \end{aligned}$$

So the linear approximation for the expected value is

22 $$\begin{aligned} \lambda ^{pS} = \lambda \psi \xi U^S + \lambda W^S, \end{aligned}$$

where $U^S$ is defined by (14) and $W^S \equiv \frac{2 \psi \xi \tau ^2}{N(t)} \left( \mu S(t) - \Lambda N(t)\right) \left( 1-3K_N(t) \tau \right)$.

If $N(t) = N_0$, then $\Lambda =\mu$. Consequently $K_N(t)=0$ by substitution into (6), and $\lambda ^{pS}$ reduces to

23 $$\begin{aligned} \lambda ^{pS}\approx & {} \frac{ \psi \xi \lambda \tau \left( S(t) \left( 1+ 2 \mu \tau \right) - 2 \mu N_0 \tau \right) }{N_0},\nonumber \\= & {} \psi \xi \lambda \tau \left( \frac{S(t)}{N_0} - 2 \mu \tau \left[ \frac{N_0 - S(t)}{N_0} \right] \right) . \end{aligned}$$

### Combining rates of infection

In this section, we combine the rates of transmission from casual partnerships with the rates of transmission from a long-term partner who was chosen while infectious $\lambda ^{pI}$, and a long-term partner who was chosen while susceptible, $\lambda ^{pS}$, but has been newly infected outside the long-term partnership. Combining (2), with (13) and (22), we have the following implicit relationship for the rate of infection, $\lambda$,

24 $$\begin{aligned} \lambda =\lambda ^z + U^I +\lambda \left[ W^S+ (\psi \xi -\chi _a) U^S\right] . \end{aligned}$$

Solving for $\lambda$ yields

25 $$\begin{aligned} \lambda= & {} \frac{z \left( \beta _a I_a+ \beta _c I_c +\beta _v I_v\right) + U^I N}{N + \left( \psi \xi -\chi _a \right) \tau S (2 K_N\tau -1) + 2 \psi \xi \tau ^2 T}, \nonumber \\ U^I N= & {} \left( \chi _a I_a+ \chi _c I_c+ \chi _v I_v \right) (1 - K_N \tau ) + \nonumber \\{} & {} \left( K_A I_a+ K_C I_c+ K_V I_v\right) \tau (1 - 2 K_N \tau ),\nonumber \\ T= & {} \left( \mu S - \Lambda N \right) \left( 3K_N \tau -1\right) . \end{aligned}$$

The definition of $\lambda$ completely specifies the problem defined by (1).

One of the items of interest is to measure how many infections are caused by casual or long-term partners in the acute class, the chronic class, and the virally suppressed class. To capture this information, we separate $\lambda$ according to casual or long-term partnerships

$$\begin{aligned}&\lambda _z^j&= \frac{z \beta _j I_j}{N + \left( 2 \psi \xi \tau ^2\right) \left( \mu S - \Lambda N \right) \left( 3K_N \tau -1\right) + \left( \psi \xi -\chi _a \right) \tau S (2 K_N\tau -1) }, \\&\lambda _{LT}^j&= \frac{\left[ \chi _j (1 - K_N \tau )+ K_j \tau (1 - 2 K_N \tau ) \right] I_j}{N + \left( 2 \psi \xi \tau ^2\right) \left( \mu S - \Lambda N \right) \left( 3K_N \tau -1\right) + \left( \psi \xi -\chi _a \right) \tau S (2 K_N\tau -1) }, \end{aligned}$$

where $j=a,c,v$ corresponding to acute, chronic or virally suppressed. The separation of $\lambda$ allows us to tract both casual and long-term susceptible populations

26 $$\begin{aligned} \frac{d S_z^j}{dt}= & {} \lambda _z^j S,\;\;\;\;\;\;\; \;\; \frac{d S_{LT}^j}{dt} = \lambda _{LT}^j S,\nonumber \\ \frac{dS}{dt}= & {} \pi - \mu S - \sum _{j=a,v,c}\left( \frac{d S_z^j}{dt} + \frac{dS_{LT}^j}{dt}\right) , \end{aligned}$$

where the three equations $d S_z^j/dt$ represents the number of susceptibles infected by a casual partner in stage $I_j$ and the three equations $d S_{LT}^j/dt$ represents the number of susceptibles infected by a long-term partner in stage $I_j$. Adding these equations to (1), we have

27 $$\begin{aligned} \begin{aligned} \frac{d S_z^a}{dt}&= \lambda _z^a S, \;\;\;\;\;\;\; \;\; \frac{d S_z^v}{dt} = \lambda _z^v S, \;\;\;\;\;\;\;\;\;\;\; \frac{d S_z^c}{dt} = \lambda _z^c S,\\ \frac{d S_{LT}^a}{dt}&= \lambda _{LT}^a S,\;\;\;\;\;\; \frac{d S_{LT}^v}{dt} = \lambda _{LT}^v S,\;\;\;\;\;\; \frac{d S_{LT}^c}{dt} = \lambda _{LT}^c S,\\ \frac{dS}{dt}&= \pi - \mu S - \sum _{j=a,v,c}\left( \frac{d S_z^j}{dt} + \frac{dS_{LT}^j}{dt}\right) , \\ \frac{dI_a}{dt}&= \lambda S - \rho I_a - \mu I_a, \\ \frac{d I_c}{dt}&= (1-\sigma ) \rho I_a +\eta I_v -\omega I_c - (\mu +\mu _c) I_c, \\ \frac{d I_v}{dt}&= \sigma \rho I_a -\eta _j I_v +\omega I_c - (\mu +\mu _v) I_v,\\ \frac{d N}{dt}&= \pi -\mu _c I_c - \mu _v I_v -\mu N, \end{aligned} \end{aligned}$$

for a total of eleven differential equations.

### Parameter values

To determine a value for $\omega$, the rate of acquiring viral suppression, we used the estimate that in 2011 median time from HIV infection to diagnosis was 3 years and 7 months, Centers for Disease Control and Prevention (2017). In 2015, median time was 3 years. Then a 2012–2017 study (Crepaz et al. 2020) measured that the median diagnosis to viral suppression interval shortened overall for persons with HIV diagnosed in 2012 vs. 2017 from 9 to 5 months. Therefore, we use $1/\omega =$ 36 months + 5 months - 3 months (time in acute phase). To most accurately capture the changes viral suppression has made over the years in the US, we would need to vary $\omega$ over time, but using a value in the middle of the 2005–2025 time line gives us a reasonable approximation.

Virological failure can have many reasons from suboptimal adherence and drug intolerance/toxicity to the high cost of HAART (Panel on Antiretroviral Guidelines for Adults and Adolescents 2021). Therefore, the value for $\eta$ is highly variable. We use a base value from Ledergerber et al. (1999); Kim et al. (2014), with the understanding a sensitivity analysis will be needed to determine how a range of $\eta$ values will affect our outcomes.

**Table 1:** Parameter descriptions, values and citations for HIV

Params	Value	Description	References
$\Lambda$	$.73\%$	Percent population growth	Estimated
$\pi$	$\Lambda N(t)$	Rate of becoming sexually	Estimated
		Active	
$\mu$	$\frac{1}{61}$/year	Natural death rate	Centers for Disease Control and Prevention (2014)
$\mu _c$	0.0219/year	HIV removal rate	Calculated
		from $I_c$	
$\mu _v$	0.003/year	HIV Removal Rate	Nakagawa et al. (2012)
		from $I_v$	May et al. (2011)
*c*	52/year	Average number sex	Estimated
		acts/year	
$\tau$	3.57 years	Long-term Partnership	Weiss et al. (2020)
		Duration	
*z*	12.05/year	Ave. number of casual	Van Tieu et al. (2014)
		Partners/year	
$p/\tau$	0.210/year	Ave. Number of long-term	Calculated
		Partners/year	
*f*	0.837/year	Pair formation rate	Weiss et al. (2020)
$\xi$	26.4%	Ave. probability of extra-	Weiss et al. (2020)
		partnership sexual act	
$1/\rho$	1/4 year	Time of transition	Fiebig et al. (2003)
		From $I_a$	Robb and Ananworanich (2016)
$$n_a $$	$$c/\rho $$	Number of exposures	Calculated
		to $I_a$ per Partnership	
$n_c$	$c \tau$	Number of exposures	Calculated
$=n_v$		to $I_c$ or $I_v$ per Partnership	
$\beta _c$	0.0075	Transmission probability	Patel et al. (2014)
		For $I_{c}$	
$\beta _a$	$10.8 \cdot \beta _c$	Transmission probability	Pilcher et al. (2007)
		For $I_{a}$	Hollingsworth et al. (2008)
			Hughes et al. (2012)
			Pilcher et al. (2004)
$\omega$	12/38	Rate of obtaining viral	Crepaz et al. (2020)
	1/year	Suppression	
$\eta$	0.235	Rate of losing viral	Ledergerber et al. (1999)
	1/year	Suppression	Kim et al. (2014)
$\sigma$	$10\%$	Percent in early viral	Estimated
		Intervention	
$\theta$	70%	Condom effectiveness	Smith et al. (2015)
$\alpha _z$	85%	Condom usage in casual	Estimated
		Partnerships	
$\alpha _p$	20%	Condom usage in	Estimated
		Long-term partnerships	

We define our number of long-term partners per year, $p/\tau$, from pair formation models. Using the definitions reviewed in Kretzschmar and Heijne (2017), *M* is the number of lifetime long-term partners, and $M = f/(\mu (f \tau + 1))$, where *f* is the pair formation rate, $\tau$ is the partnership duration, and $1/\mu$ is the lifetime. So the number of long-term partners per year is $M \mu = f/(1+f \tau )$, which is our definition for $p/\tau$ (Gurski et al. 2023). In Gurski et al. (2023) it was shown that with these definitions, the long-term partnership model matches the behavior of the pair formation model with both reproduction numbers and numerical simulations in time.

We follow the work of Hyman et al. (2001) to describe the transmission rates $\chi _a$, $\chi _c$, $\chi _v$, and $\psi$. The term $\chi _j$ is the transmission rate by a partner in the infected class $I_j$ with $j = \{a,c,v\}$. Just as with the casual sexual partnership infection term, the infected partner in $I_j$ can possibly infect the susceptible partner in a single sexual act at a probability of $\beta _j$ mitigated with condoms. We introduce the transmission factor term due to condom use, $\theta \alpha _z$ for a casual partnership and $\theta \alpha _p$ for a long-term partnership. The term $(1-\theta \alpha _x) \beta _i$ is the transmission per sexual act ($x =\{z,p\}$), that represents the reduction from condom effectiveness and usage. The probability of not being infected in a single act is then $(1-(1- \theta \alpha _x) \beta _j)$. So the probability of not being infected after $n_j$ sexual acts with the $I_j$ partner is $(1- (1-\theta \alpha _x) \beta _j)^{n_j}$. We assume 85% condom usage in the casual pairs but only 20% use in the long-term partnerships. The probability that the susceptible long-term partner will be infected after $n_j$ ($j = \{a,c,v\}$) sexual acts with the long-term partner in $I_j$ is $\chi _j = (p/\tau ) \left( 1- (1-(1-\theta \alpha _p) \beta _j )^{n_j} \right)$, where the term $p/\tau$ is the number of long-term partners per year, i.e. the rate of acquiring long-term partners. The exponent $n_j$ reflects the number of exposures over the duration of the partnership while the partner is in the infection class *j*. That is, during the acute phase, $n_a = c/\rho$, and during the chronic or virally suppressed stage, $n_c=n_v = c \tau$. We assume that the transmission rate by a newly infected long-term partner is $\psi = (p/\tau ) \beta _a$. Since both partners were formerly both susceptible, we assume that the long-term partners are not using condoms with each other. With an estimate of 1 sex act per week, there will be 9 contacts to transmit HIV during this highly infectious acute stage, before either partner has been tested and started taking active precautions to safeguard their partner.

## Results

The results of this model were all simulated beginning with the initial conditions given by the CDC data in 2005 (Centers for Disease Control and Prevention 2012b) and the parameters given in Table 1. We begin with the reproduction number of the long-term and casual model in Sect. 4.1 and demonstrate how the behavior of the reproduction number depends on the various parameters in the model. Next, in Sect. 4.2, we compare incidence and prevalence model data to CDC data to illustrate validation of the proposed model. For varying values of $\eta$ and $\omega$, time series simulations with both the model that includes both long-term and casual partnerships and the model with only long-term partnerships show the effect of achieving and remaining in viral suppression. There has been much speculation on the role of the acutely infected population in the spread of HIV. We address this concern in Sect. 4.3 both as a function of infectivity and whether the encounter was from a casual partnership or a long-term partnership. Finally, we address the sensitivity of the parameters on prevalence of HIV in Sect. 4.5

### Reproduction number

The effective reproduction number can be analytically determined using the standard Next Generation methods (Diekmann et al. 1990; van den Driessche and Watmough 2002):

28 $$\begin{aligned} {\mathcal {R}}_e= & {} \frac{A ( F+ \beta _a z) + C \rho (\chi _c + \beta _c z) + B \rho (\chi _v + \beta _v z)}{A (\mu + \rho ) (1 - D \tau ) E}, \end{aligned}$$

with parameters

29 $$\begin{aligned} \begin{aligned} A&= (\mu +\mu _c) (\mu + \mu _v + \eta ) + (\mu + \mu _v) \omega ,\\ B&= \omega + (\mu +\mu _c) \sigma ,\\ C&= \eta + (\mu + \mu _v) (1-\sigma ), \\ D&= \chi _a (1 + 2 (\Lambda -\mu ) \tau )-(1 - 6 (\Lambda -\mu )^2 \tau ^2) \psi \xi ,\\ E&= 1 - \chi _a (1+ 2 (\Lambda -\mu ) \tau )\tau ),\\ F&= \chi _a (1+ (\Lambda -\mu ) \tau -D (\mu + \rho ) \tau ^2 (1 + 2 (\Lambda -\mu ) \tau ). \end{aligned} \end{aligned}$$

The reproduction number depends on several parameters, many nonlinearly. We investigate the effects of each parameter on the reproduction number so we may identify interventions that mitigate the effects of parameters that increase the reproduction number and interventions that support the effects of the parameters that lower the reproduction number.

While the expression for ${\mathcal {R}}_e$ is algebraically complex, *z* appears linearly in the numerator, therefore ${\mathcal {R}}_e$ is a linearly increasing function of *z*, the rate of the number of casual partners per year. The parameter $\sigma$ appears in *B* and *C* linearly. Since $\chi _v < \chi _c$ and $\beta _v < \beta _c$, ${\mathcal {R}}_e$ decreases linearly as a function of $\sigma$, the percentage of acutely infected individuals that go directly to the virally suppressed category. The effective reproduction number, ${\mathcal {R}}_e$, is linearly increasing as a function of the number of sex acts per year, *c*, since $\chi _i \approx p\, (1-\theta \alpha _p) \, \beta _i \,c$ and

$$\begin{aligned} C \rho (\chi _c + \beta _c z) + B \rho (\chi _v + \beta _v z) \approx \rho (C \beta _c + B \beta _v) (p c (1-\theta \alpha _p) + z (1-\theta \alpha _z)). \end{aligned}$$

**Fig. 2:**
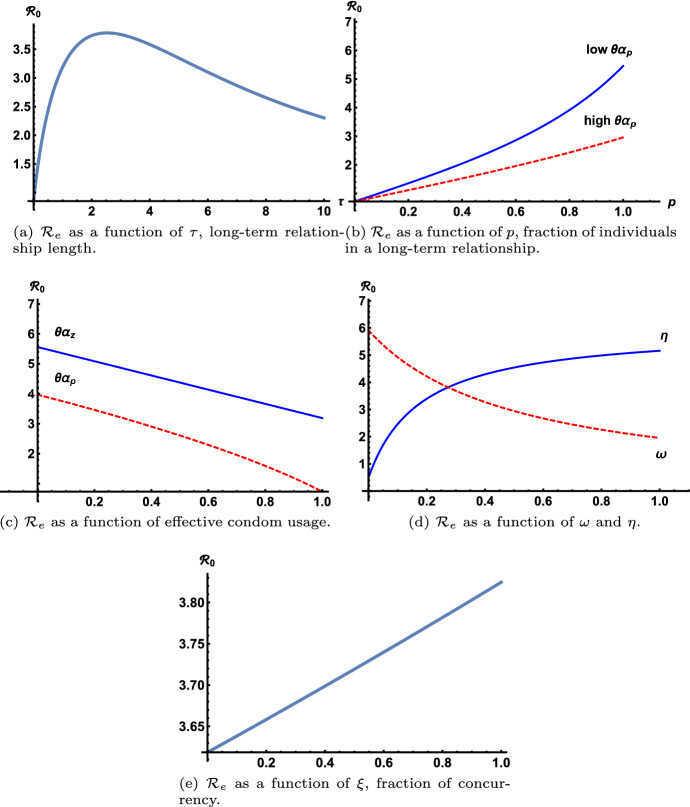
The effective reproduction number is plotted as a function of the variables $\tau , p, \theta \alpha _z, \theta \alpha _p, \eta , \omega , \xi$, with parameters at fixed values found in Table 1, except for the parameter that is varied in each graph (color figure online)

The relationship of ${\mathcal {R}}_e$ with parameters $\tau , p, \theta \alpha _z, \theta \alpha _p, \eta , \omega , \xi$ is shown graphically in Fig. 2. Figure 2a shows that ${\mathcal {R}}_e$ increases as a function of $\tau$ until around $\tau =3$ years, then ${\mathcal {R}}_e$ decreases, which implies that longer relationships reduced the value of ${\mathcal {R}}_e$. As the fraction of MSM in long-term partnerships grows, *p*, ${\mathcal {R}}_e$ grows, but the rate of growth depends on the condom use in long-term partnerships. In Fig. 2b the curve marked “low $\theta \alpha _p$” refers to the 20% condom usage with 70% condom effectiveness, the curve marked “high $\theta \alpha _p$” refers to the 85% condom usage with 70% condom effectiveness. The “high $\theta \alpha _p$” value was chosen to match condom effectiveness in casual sex partnerships, $\theta \alpha _z$. We see that if effective condom usage is increased in the long-term partnerships, then ${\mathcal {R}}_e$ grows much slower as the fraction of long-term partnerships increases.

Figure 2c shows the inverse relationship of ${\mathcal {R}}_e$ as a function of effective condom usage for both casual partnerships, solid blue line and the long-term partnerships, dashed red line. As more individuals transition from chronically infected to virally suppressed, the values of the effective reproduction number decreases shown by the red dashed line in Fig. 2d and as individuals leave the virally suppressed group and transition back to chronically infected, ${\mathcal {R}}_e$ increases, as shown by the solid blue line in Fig. 2d. Figure 2e illustrates the growth of ${\mathcal {R}}_e$ as the measure of concurrency in long-term partnerships, $\xi$, increases. Based on these results, effective migitation strategies should include more long-term relationships with longer than average relationship length, more effective condom usage and less concurrency in the long-term partnership. Additionally, the more individuals we can recruit and keep in the virally suppressed stage, the more the reproduction number will decrease.

### Incidence and prevalence

**Fig. 3:**
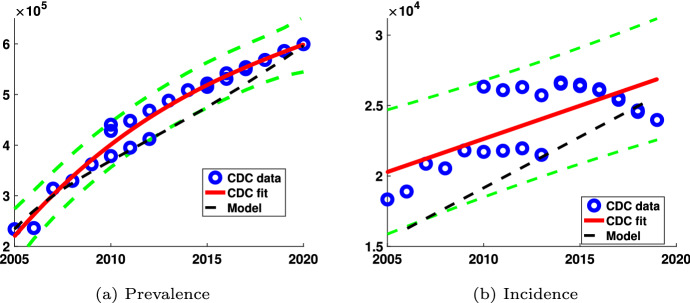
The HIV incidence and prevalence are plotted as a function of the year between 2005 and 2020 for the model that includes both casual and long-term partnerships. The blue dots are data from the CDC. The black dashed line corresponds to model output and the solid red line corresponds to the linear (Incidence) and quadratic (Prevalence) regression fits to the CDC data over this time interval. The green dashed curves correspond to the 95% confidence interval (color figure online)

Figure 3 shows the incidence of new infections and prevalence of HIV infections each year from 2005 to 2020 for MSM in the U.S. with both long-term and casual partnerships included in the model along with CDC incidence and prevalence data. As we have illustrated in Fig. 3, our model closely approximates the incidence and prevalence of HIV infections in the US in from the 2005–2019 data. HIV incidence and prevalence data from the CDC HIV Surveillance reports, HIV Surveillance Supplemental Reports, and prior to 2008 the HIV/AIDS Surveillance reports (Centers for Disease Control and Prevention 2022a) are shown using the blue circles. Data for each year represents the multiple years that the CDC updates its data estimates, hence the multiple data points for some years. The solid red curve is a curve fit to CDC data and the green dashed lines represent a 95% confidence interval for the data. The black dashed line in the prevalence graph Fig. 3a represents the model output for prevalence. The model output for incidence was determined by fitting the model prevalence output to a quadratic curve (not shown). The derivative of the quadratic curve at each year represents the HIV incidence for that year and is illustrated as the dashed black line in the incidence graph. Our prevalence and incidence are within the 95% confidence interval of the CDC data.

**Fig. 4:**
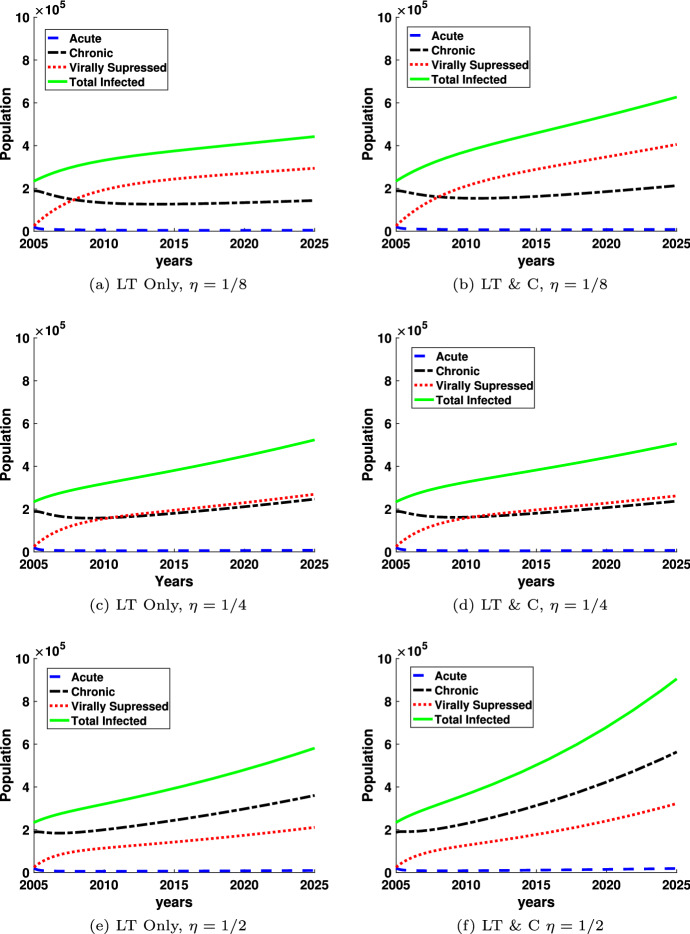
HIV Prevalence is plotted as a function of year between 2005 and 2025 for varying values of $\eta$, the rate at which virally suppressed individuals lose suppression and transition to chronically infected, with two different models: one with long-term monogamous partnerships (LT Only) and one with both long-term and casual partnerships (LT & C). All other parameters are held at base values found in Table 1 (color figure online)

**Fig. 5:**
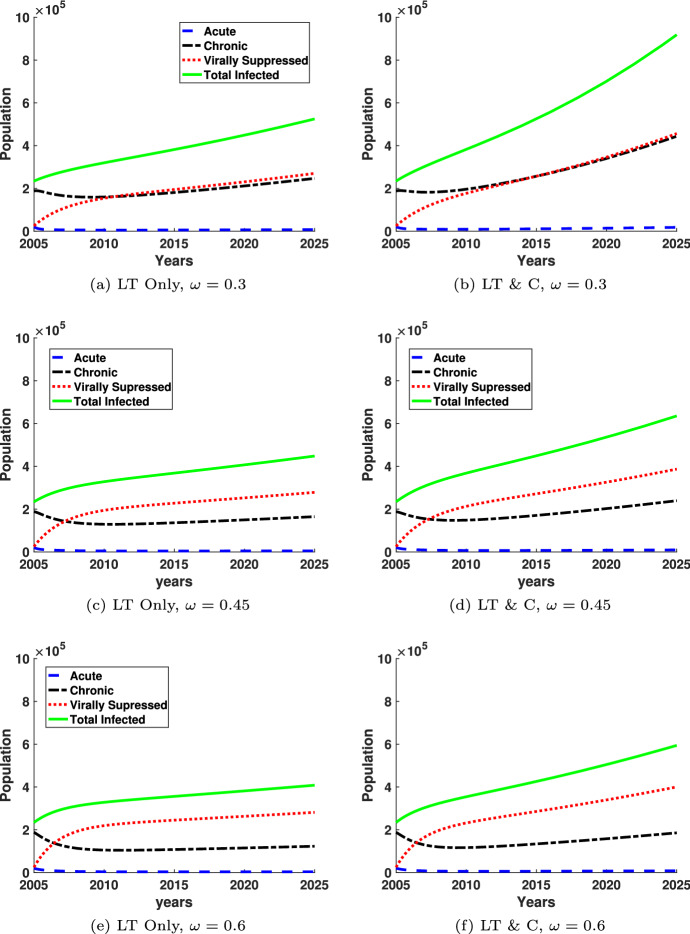
HIV Prevalence is plotted as a function of year between 2005 and 2025 for varying values of $\omega$, the rate at which chronically infected individuals gain suppression and transition to virally suppressed, and two different models: one with long-term monogamous partnerships (LT Only) and one with both long-term and casual partnerships (LT & C). All other parameters are held at base values found in Table 1 (color figure online)

Figures 4 and 5 show the prevalence of HIV infected individuals and the behavior of the different infected populations over time for both the monogamous long-term partnership model (LT Only) and the model with both long-term and casual partnerships (LT & C). In both figures, the population of each infected class is shown as a function of the years between 2005 and 2025. The green solid line corresponds to the sum of all the infected individuals. The red dotted line corresponds to the virally suppressed population and the black dash-dotted line corresponds to the chronic class of infected. The acute class of infected is shown by the blue dashed line. The initial conditions for the simulations correspond to the data reported by the CDC (Centers for Disease Control and Prevention 2012b) in 2005. Although the acute class of infected appears very small in these plots, there are roughly 1,000–2,000 cases at any given time which is about 1/10th of the total number of HIV infections.

Figure 4 plots the population of each class of the infected population from 2005 to 2025 for four different values of $\eta =1/8, 1/4, 1/2$, the rate at which the virally suppressed population loses suppression and transitions to the chronic population, for both the long-term monogamous model and the model with both long-term and casual partnerships. For all graphs the number of acutely infected individuals is small compared to the other populations because acutely infected individuals transition quickly, relative to the timescale, to the chronic or virally suppressed populations. The relationship between the chronically infected population and the virally suppressed population changes as a function of $\eta$. As $\eta$ increases, the virally suppressed population decreases, as illustrated by the red dotted line, and the chronically infected population (black dash-dot line) increases as a function of $\eta$. For the long-term monogamous partnership model, as seen in Fig. 4a, $\eta =1/8$, the virally suppressed population is above the chronically infected population after about 2008. This trend is approximately the same for Fig. 4c. However, for $\eta =1/2$, the chronic population (black dashed-dotted line) is higher than the virally suppressed population for the entire time interval shown. Similar patterns are evident for both the long-term monogamous model and long-term and casual partnership model, although the effects seem to be amplified slightly for the long-term model with casual partnerships.

Figure 5 shows the same graphs as Fig. 4, except $\omega$ is changing instead of $\eta$. The parameter $\omega$ represents the rate of transition from the chronically infected class to the virally suppressed class. In Fig. 5, the virally suppressed class is above the chronically infected class for most of the time interval shown. The relationship between the virally suppressed class the chronically infected class changes as $\omega$ increases and more individuals achieved suppression and are moved from the chronic infected class to the virally suppressed infected class. Since treatment reduces the rate of infectivity compared to the chronic class of infected, if enough of the infected population is in treatment, the prevalence of infection will eventually decrease, as we see for $\omega$ sufficiently large in both models of long-term and casual partnerships and monogamous long-term partnerships.

### Infections transmitted from partner in class $I_a$, $I_c$, or $I_v$

Susceptible individuals can become infected from either a long-term partnership or a casual partnership, and the infected partner can be in one of the three stages of infection: acute, chronic, and virally suppressed. While the acute class is the most transmissive stage of infection, the length of time an individual is in the class is much smaller compared to the time that individuals spend in either chronically infected or virally suppressed. There as been much scientific debate on which class of infection contributes more to the spread of HIV.

**Fig. 6:**
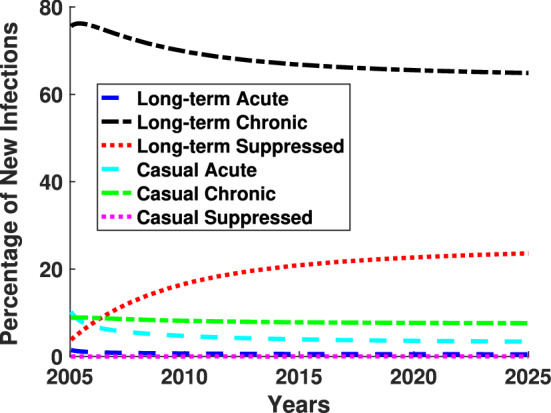
Percentages of new infections due to partner in particular infected class for each year are illustrated for baseline parameter values found in Table 1. The different linestyles and colors indicate the three stages of infection and two types of partnership models, as shown in the legend (color figure online)

To address this question, we separately track the number of susceptible individuals that are infected by acute, chronic, or virally suppressed individuals by splitting the force of infection term into acute, chronic, and virally suppressed for both casual and long-term partnerships, as described by system (27). Figure 6 illustrates the percentage of incident infections from the six possible infected types: casual or long-term partner, in one of three stages of infection, for baseline parameter values found in Table 1. The percentage of susceptible individuals that are infected by a long-term partner is represented by the solid blue line for those in the acute stage of infection, the dot-dashed black line for those in the chronic state of infection, and the dotted red line for those in the virally suppressed stage of infection. Similarly, the percentage of susceptible individuals infected by a casual partner is represented by the solid cyan line for those in the acute stage of infection, the dot-dashed green line for those in the chronic state of infection, and the dotted pink line for those in the virally suppressed stage of infection. Most of the new HIV infections are from chronic long-term partners with casual partnerships, with long-term acute and casual virally suppressed partners contributing the least to new infections.

**Fig. 7:**
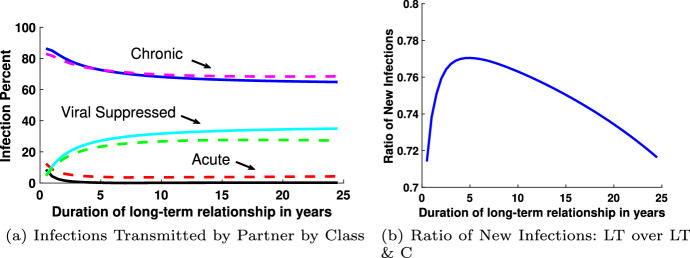
New infections in 2015 as a function of the duration of the long-term-relationship. The solid line in **a** represents long-term monogamous partnerships only, the dashed line represents the long-term and casual partnerships (color figure online)

Additionally, by tracking new infections transmitted by the type of partner in a particular stage we are able to address the number of infections transmitted by a partner in the acute stage. We illustrate the number of new infections as a function of the duration of the long-term partnership, similar to Kim (2015). As we have illustrated in Fig. 3, our model closely approximates the incidence and prevalence of HIV infections in the US in 2015, hence we present our results with respect to 2015. Figure 7a illustrates the percentage of infections transmitted by a partner in the acute class in 2015, the chronic class in 2015, and the virally suppressed class in 2015, as a function of the duration of the long-term relationship. The solid lines indicates results from the long-term monogamous relationship model and the dashed lines show the results for the model that includes both long-term and casual partnerships. After 5 years, the model with both long-term and casual partnerships has a higher percentage of infections than the long-term monogamous model transmission for both the acute and chronically infected individuals. However, in transmission from the virally suppressed individual, the percentage of infections from the long-term monogamous model is larger than the model that includes both long-term and casual partnerships since the long-term monogamous partners are only exposed by way of a long-term partner in the virally suppressed class. This may seem unexpected, but the number of new infections in the long-term monogamous model is significantly fewer than the number of new infections in the long-term plus casual partnership model. The blue curve in Fig. 7b shows a decreasing ratio of incident infections after five years from the long-term monogamous partnership model as compared to the long-term and casual partnership model over the increasing length of the long-term partnership. This figures shows the “protective” effect of a monogamous long-term relationship against HIV. The drop in the number of new infections is not due to a much larger number of sexual acts in the long-term and casual group. Instead, this effect is due to potential overlap or concurrency of sexual partnerships driving up the number of transmissions.

Overall, transmission from the chronically infected individual contributes significantly more to the spread of new infections than either an acutely infected or virally suppressed individual, similar to the results shown in Fig. 6. We note that while the prevalence of people with acute HIV infections appears insignificant in Figs. 4 and 5, the impact of these individuals in the acutely infected class is significant as shown in Fig. 6 and 7. The impact of the infections transmitted by the individuals in the acute HIV infection class is also increased when relationships are non-monogamous and allow an overlap of partnerships, i.e. concurrent sexual relationships.

### Effective condom usage

**Fig. 8:**
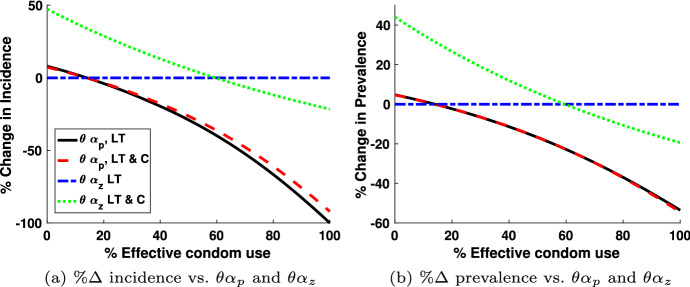
The percentage change in new infections due to increased effective condom usage ($\theta \alpha _p$) in long-term partnerships and casual partnerships ($\theta \alpha _z$) are shown for both the long-term monogamous model and the model that includes both long-term and casual partnerships in **a**. The percentage change in prevalence is shown in **b**. The baseline value for $\theta \alpha _p$=14% and $\theta \alpha _z$ = 59.5% (color figure online)

Figure 8 examines the impact of the parameters $\theta \alpha _p$ and $\theta \alpha _z$ on the percentage change in incidence and percentage change in prevalence, for both long-term monogamous model and the model that includes both long-term and casual partnerships. The percentage change in incidence is computed by dividing the difference of the incidence with the baseline parameters given in Table 1 and the incidence computed with the percentage on the *x*-axis by incidence at the baseline parameters. Figure 8a illustrates the percentage change in incidence of new infection as a function of the parameters relative to effective condom use in long-term partnerships ($\theta \alpha _p$) and effective condom use in casual partnerships ($\theta \alpha _z$) in the year 2015 (10 years after the start date of our simulation). The black solid curves represent the change in $\theta \alpha _p$ for long-term monogamous partnerships, and the red dashed curves represent the change in $\theta \alpha _p$ for long-term and casual partnerships. The blue dash-dot curves represent the change in $\theta \alpha _z$ for long-term monogamous partnerships, and the green dotted curves represent the change in $\theta \alpha _z$ for long-term and casual partnerships.

As expected, the curves in Figs. 8a and 8b, decrease as effective condom use increases. The magnitude of the percentage decrease is different in each case. Figure  8 shows little difference in the effect of increased effective condom use in long-term partners between models (red dashed line and solid black line), however the magnitude of the decrease is significant indicating that this intervention has potentially a large impact on HIV infection incidence. Note that the base effective condom use for long-term partnerships is assumed to be quite low, $\theta \alpha _p = (0.70)(0.2) = 14\%$. Increased effective condom usage with casual partnerships has no effect on the long-term monogamous model, as indicated by the horizontal lines in Figs. 8a, b, however, significantly impacts the model that includes both long-term and casual partnerships. While the range of percent change in incidence is large, Fig. 8 does not show a decrease in percent change in incidence for varying $\theta \alpha _z$ until approximately $\theta \alpha _z = (0.70)(0.85) \approx 60\%$, effective condom usage in casual relationships since this is the base parameter value. This pattern appears in both the incidence, Fig. 8a and prevalence, Fig. 8b.

### Parameter sensitivity

**Table 2:** Parameter values and ranges for LHS-PRCC

Parameter	Value range	Baseline
$\mu$	(1/70,1/50)	1/61
$\mu _c$	(0.01,0.03)	0.291
$\mu _v$	(0.002,0.007)	0.003
$\sigma$	(0,.2)	0.10
$\xi$	(0.1,0.6)	0.264
$\tau$	(0.05,10)	3.57
*z*	(2,40)	12.05
*p*	(0.1,1)	0.749
$\eta$	(0,0.4)	0.235
$\omega$	(0.2,0.8)	0.316
$\theta \alpha _z$	(0,0.7)	0.595
$\theta \alpha _p$	(0,0.3)	0.14
*c*	(26,104)	52

**Fig. 9:**
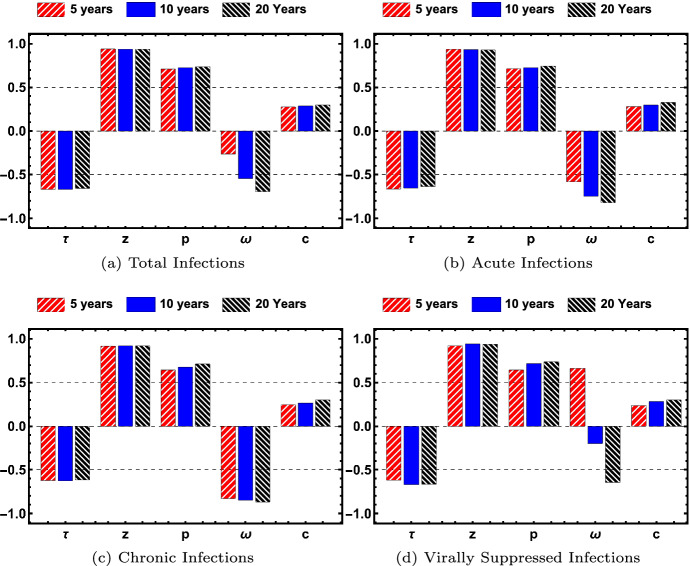
The partial rank correlation coefficient is shown for each of the parameters $\tau , z, p, \omega , c$ for total number of acute, chronic and virally suppressed individuals. Results shown are significant with *p*-value < 0.05 (color figure online)

**Fig. 10:**
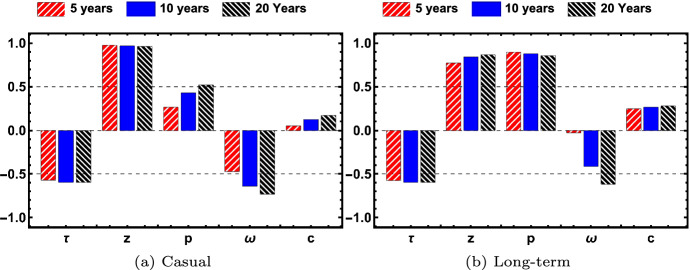
The partial rank correlation coefficient is shown for each of the parameters $\tau , z, p, \omega , c$ for infections caused by a casual or long-term partner. Results shown are significant with *p*-value < 0.05 (color figure online)

We consider the parameter sensitivity of transmission by infected type (acute, chronic, virally suppressed) in Fig. 9 and by partnership model (long-term monogamous and long-term and casual) in Fig. 10 to all the model parameter values in the specified range given in Table 2, using Latin Hypercube Sampling (LHS) and Partial Rank Correlation Coefficients (PRCC). The magnitude of the PRCC indicates the strength of the correlation between the parameter and the output, whereas the sign of the PRCC indicates whether there is a positive or negative correlation between the parameter and the output. Sensitivity analysis is important for determining which parameters have the largest impact on the dynamics of the spread of the infection. Figures  9 and 10 show the values of the PRCC for the parameters $\tau , z, p, \omega , c$ at the time intervals of 5 (red, upward hash), 10 (blue, solid), and 20 (black, downward hash) years after 2005 for prevalence and incidence, respectively. Only significant results with *p*-value < 0.05 are shown.

Figure 9 shows the PRCC values for the total number of infections in Fig. 9a, the number of acute infections in Fig. 9b, the number of chronic infections in Fig. 9c, and the number of virally suppressed infections in Fig. 9d. In all figures, the parameter *p* is strongly positively correlated with the number of infections, which means as the parameter value increases, the number of infections increases. The average number of sex acts per year *c* and the average number of casual partner *z* are also positively correlated with the number of infections. The average length of a long-term relationship, $\tau$, is negatively correlated with the number of infections, which means that longer relationships decrease the number of infections. These correlations are (mostly) independent of time, since the magnitude does not change significantly over 5, 10, or 15 years. The PRCC values for the parameters associated with transitioning to the virally suppressed $\omega$ vary over time and even change sign of correlation. The rate at which the chronically infected transition to virally suppressed $(\omega )$ is negatively correlated with total, acute, and chronic infections, with the largest impact on the prevalence of chronically infected. The parameter $\omega$ is strongly positively correlated with virally suppressed at 5 years, weakly positively correlated at 10 years and strongly negatively correlated at 20 years. This effect is seen in Fig. 5 where the steepest growth of the virally suppressed class in in the five years.

Figure 10 shows the sensitivity to new infections caused by long-term or casual by partnership type, where Fig. 10a corresponds to new infections caused by casual partnerships and Fig. 10b corresponds to new infections caused by long-term partnerships. As with the previous figure, $\tau$, average relationship length is negatively correlated with number of infections and *z*, *c*, *p* are positively correlated with number of infections, with *p* more positively correlated with new infections from long-term partnerships and *z* more positively correlated with casual partnerships. The parameter $\omega$ is negatively correlated but the correlation grows over time for both long-term and casual partnerships.

The effect of early intervention, the percentage $\sigma$, effective condom usage in casual encounters, $\theta \alpha _z$, and effective condom usage in long-term partnerships, $\theta \alpha _p$, are not significant in the sensitivity analysis. It appears from Fig. 8 that $\theta \alpha _p$ should be significant, but not when keeping realistic effective condom usage in long-term partnerships. In keeping with the disinclination to use condoms in the long-term partnerships, we test only for sensitivity with effective condom usage in the range of 0% to 30% as shown in Table 2. Also $\theta \alpha _z$ should be significant as well, but we have already assumed a base of 85% use with a 70% per use effectiveness for receptive anal sex, so this reduces the sensitivity of the parameter $\theta \alpha _z$ to the number of infections.

### Early intervention

It has been shown that in addition to prolonging life and the quality of life, there are significant societal economic benefits to the early diagnosis of HIV (Department of Health and Human Services 2022). While HIV screening is a key step to this, the standard test for HIV cannot detect infection during acute stage. However, there are small clinical studies being done to test for HIV in the acute stage (Granich et al. 2009; Xiridou et al. 2003; Lindback et al. 2000; Ananworanich et al. 2015; Ford et al. 2018). We use our mathematical model to investigate the benefits of early intervention if a readily available and easy-to-use test were to exist and were used extensively.

**Fig. 11:**
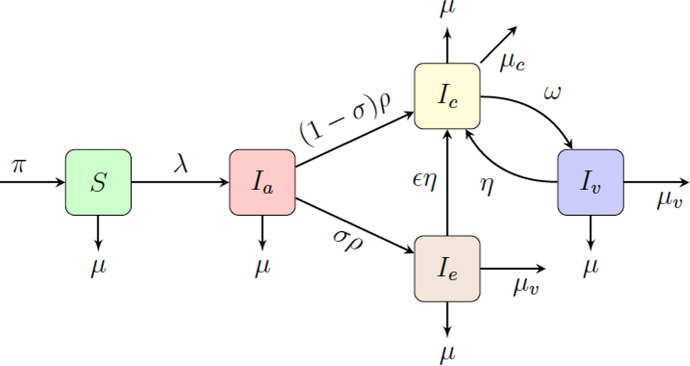
$$S I_a I_c I_e I_v $$ model where *S* is the susceptible population, $$I_a$$ is the acutely infected population, $$I_c$$ is the chronically infected and not virally suppressed population, $$I_e$$ is the infected and early virally suppressed population, $$I_v$$ is the infected and virally suppressed population

**Fig. 12:**
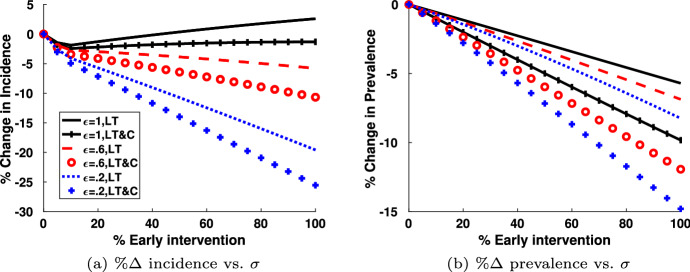
**a** and **b** Illustrate the change in incidence and prevalence as a function of increasing early treatment to the change in $\sigma$, the percentage of successful early interventions. The parameter $\epsilon$ refers to the loss of viral suppression at a rate of $\epsilon \eta$, from this early intervention viral suppressed stage

The parameter $\sigma$ corresponds to the percentage of acutely infected individuals that also have early intervention and viral suppression. By introducing the acute class, $I_a$ into our model, we can investigate the effect of early intervention by diagnosing HIV in the acute stage rather than the chronic stage. If this test were available, not only would it help move HIV positive individuals to viral suppression faster, the individuals could be treated before their healthy CD4 cell counts drop significantly. The magnitude of the CD4 cell recovery has been shown to be directly correlated with the CD4 count at the initiation of highly active anti-retroviral therapy (Department of Health and Human Services (2022)). So we suggest a modification to our model that might capture the higher CD4 count from early intervention. The flow chart for this model is shown in Fig. 11. In this model we add a separate category $I_e$ where those individuals with acute HIV were discovered at a percentage $\sigma$ and were given early intervention therapy leading to viral suppression. If those individuals have a higher CD4 cell count, they may lose viral suppression at a rate lower than $\eta$. We denote this rate of the loss of viral suppression in this early intervention category as $\epsilon \eta$, where $\epsilon$ varies between 0 and 1. The only entry to the state $I_e$ is through early testing and suppression. Once viral suppression is lost, the individuals move to the chronic state, $I_c$. If virally suppressed again, they move to the state $I_v$. With all the ODEs and definitions for $\lambda$ suitably updated, we present our theoretical results in Fig. 12. In keeping with our earlier simulations, we present results in the year 2015 (10 years after the start date of our simulation).

If viral suppression is not lost at a lower rate, i.e. $\epsilon \eta = \eta$ for $\epsilon =1$, in Fig. 12a there is no noticeable lowering in the change of incidence after 10 years for with the long-term monogamous partnership model (solid black curve) or the long-term and casual partnership model (black curve with upright hatches). We do see a change in prevalence at a maximum of −4% and −8% with full successful early intervention, see Fig. 12b, but there is no reduced rate of suppression lost for these models. However, for viral suppression lost at a 60% reduction in $I_e$ as compared to $I_v$, this effect is represented by the red dashed curve for the long-term monogamous partnership model and the red circles for the long-term and casual partnership model. In the case where $\epsilon =.6$, both the percent change of incidence and prevalence show considerable reductions. This result is magnified for viral suppression for those individuals in $I_e$ is lost at 20% the rate of viral suppression loss for those in $I_v$. The results for $\epsilon =.2$ is illustrated by the blue dotted curve for the long-term monogamous partnership model and the blue pluses for the long-term and casual partnership model. The graphs illustrate a large reduction in incidence and prevalence for $\epsilon =.2$, even for low $\sigma$. Thus the potential for this intervention is significant.

## Discussion

Modeling an MSM population, we considered a staged HIV infection model that includes susceptible, acutely infected, chronically infected, and virally suppressed individuals with both casual and long-term partnerships including concurrent partnerships. Long-term partnerships play a significant role in the spread of HIV. In contrast to previous literature, the effect of long-term partnerships on the rate of infection is captured by a linearized expected value calculation. In Gurski et al. (2023), we compared the pair formation model with the long-term partnership model. Without concurrency, the long-term partnership model and the pair formation models are almost identical, as is the reproduction number of both models. The long-term partnership model has fewer equations than the pair formation models, provides comparable model dynamics, and can more easily incorporate concurrency within the model.

We carefully analyzed the impact of each parameter on the reproduction number, the incidence (new infections), and the prevalence (total infections). The reproduction number increases as concurrency, number of casual partnerships per year, and the rate of loss of viral suppression increases. Increasing effective condom usage and transition rates to treatment decreases the reproduction number. Thus, we suggest intervention strategies that promote increasing the number and retention of viral suppression. Infections from long-term chronically infected partners are associated with the highest incidence (58%) of new infections whereas infections from casual partnerships with virally suppressed individuals contributed only 1% of the new cases. The average length of the long-term partnership is inversely related to new infections and the ratio of new infections from long-term partnerships to long-term and casual partnerships decreases as a function of relationship length, which illustrates the diminishing risk of HIV infection in long-term monogamous relationships (see Fig. 7).

Figure 3 shows a comparison of our model predictions and CDC data as a means of validating our model. Our model approximates well the CDC incidence data (Fig. 3b) within the 95% confidence interval of the CDC data. The difference between the model predictions and CDC data can be attributed to several confounding factors. We assumed constant parameter values between 2005 and 2025. The rate of gaining viral suppression, the rate of losing viral suppression, early treatment intervention changed during that time period as HAART treatment became more accessible. Additionally, in 2012, the FDA approved a preventative HIV medication regimen, the daily oral antiretroviral pre-exposure prophylaxis (PrEP) (Centers for Disease Control and Prevention 2012a) that impacted infection rates.

Acutely infected individuals are the most transmissible, but the time in the acute stage of infection is much shorter than the time in the chronic or virally suppressed stage of infection. As a result, there has been much speculation on the role of the acutely infected population plays in the spread of HIV. Figure 6 illustrates that while the long-term chronic partnerships are related to the highest incidence, acutely infected long-term partnerships contribute 10% of new infections and casual partnerships with acutely infected individuals contribute 9% of new infections. Our results are consistent with (Xiridou et al. 2004) who predicted 6% of new infections come from acutely infected steady partners using a pair formation model with moment closure. Thus, while almost 20% is a much small contribution of incidence from the acutely infected population compared with the 70% (58% long-term $+$ 12% casual) from chronically infected individuals, we note that the numbers of individuals in the acutely infected population is much smaller than the chronically infected due to the transitionary nature of the stage of infection. This is reflected in Figs. 4 and 5, where the acutely infected are 1/10th of the chronically and virally suppressed populations.

Effective condom use has been an intervention strategy to mitigate the spread of HIV for decades. Our model considered effective condom use in long-term partnerships and casual partnerships separately by assuming 85% condom usage for casual partnerships and 20% condom usage in long-term partnerships. Figure 2c shows the reproduction number decreases with effective condom usage for both long-term and casual partnerships. Figure 8 shows decreased total incidence and prevalence as a function of effective condom use for both casual and long-term partnerships. The risk of infection from long-term partners can be mitigated by effective condom use. Our model predicts that new infections from long-term partnerships from all classes of infectivity to be approximately 78%, which is consistent with results from Xiridou et al. (2003) who predicted range of 74–90%. Thus targeting effective condom use in long-term partnerships can have a potentially large impact. We note that in a stochastic pair formation model of MSM in Sweden (Hansson et al. 2019), effective condom usage of above 50% in steady partnerships and 60% in casual partnerships was found to be necessary to drive ${\mathcal {R}}_e <1$. We note that Sweden was the first country to achieve and surpass the UNAIDS/WHO 90-90-90 goal (Gisslén et al. 2017) with 90% of people living with HIV being aware of their HIV status, 95% of HIV diagnosed individuals are on HAART treatment, and 95% of those on HAART treatment are under viral suppression. Still even though Sweden is in a significantly different position in controlling HIV than the U.S., effective condom usage is equally important. Hence, our third intervention strategy promotes effective condom usage in long-term partnerships. Parameter sensitivity also shows a negative correlation between prevalence of infection for all classes (acute, chronic, virally suppressed and total) and partnership models (LT and LT & casual) for effective condom use in casual encounters. We hypothesize that condom effectiveness with long-term partners was not a parameter with a significant impact because we assume only 20% condom usage with long-term partnerships, whereas we assumed 85% usage for casual partnerships, consistent with other works (see Xiridou et al. 2003, for example).

While early detection of HIV in the acute stage of infection is unusual, we include early treatment in our model since it has been suggested as an intervention (Granich et al. 2009; Xiridou et al. 2003; Lindback et al. 2000; Ananworanich et al. 2015; Ford et al. 2018). We represent early treatment with the parameter $\sigma$, the fraction of the acutely infected population that transition directly to the virally suppressed population rather than transitioning to the chronic stage of infection. Figure 12 shows the reduction in total new infections and total infections, respectively, as more acutely infected individuals transition to the virally suppressed class. Xiridou et al. (2003) considered the reduction in infectivity during the chronic phase as a result of HAART initiated during acute stage and reported that 70–85% increase in HAART administration is beneficial even considering the increase in infectivity due to risky behavior from insufficient effective condom usage. We note that the model of Xiridou et al. (2003) does not contain a virally suppressed class, as we present here. Additionally, our result for the reduced prevalence of HIV in MSM given increased testing and early HAART treatment mirrors that of Brogan et al. (2019), although their definition of early intervention differs from ours. While accurate acute stage HIV tests are not widely available, small clinical trials are in progress. We investigated the effect of early intervention in Fig. 12. Our results indicate that more information is needed on whether viral suppression is lost at a lower rate when the HIV infection is captured and treated at the acute stage. If the rate of viral suppression lost is significantly reduced, then early intervention in the acute stage of HIV might be significant in controlling the spread of HIV.

Incorporating long-term partnerships with casual partnerships into a staged-disease model that includes acute, chronic, and virally suppressed provided a framework for three intervention strategies. First, early intervention of acutely infected individuals has a significant impact on the incidence of new infections from those acutely infected. Second, our model demonstrated achieving and maintaining viral suppression impacts total prevalence of infection in the population for both the model with long-term monogamous partnerships and long-term partnerships with casual partnerships. Finally, focusing on effective condom use in long-term partnerships, where traditionally behavioral disinhibition has kept effective condom use lower than casual partnerships, has the potential to significantly change the disease progression. Thus, the model suggests that these strategies are most impactful to reduce incidence and prevalence of HIV within the MSM population, to achieve the UNAID goal of 95-95-95 by 2030.

## Declarations

### Data and Code

Time series simulations were performed numerically with MATLAB. The analysis for the reproduction number was computed with Mathematica. The parameter sensitivity was computed using the statistical package in Matlab. The codes can be accessed upon request.
